# Draft Genome Sequence of *Mycobacterium peregrinum* Isolated from an HIV-Positive Patient in South Africa

**DOI:** 10.1128/genomeA.00759-17

**Published:** 2017-08-03

**Authors:** Shaheed V. Omar, Mushal Allam, Lavania Joseph, Senzo Mtshali, Nazir A. Ismail, Arshad Ismail

**Affiliations:** a Centre for Tuberculosis, National Institute for Communicable Diseases, National Health Laboratory Service, Johannesburg, South Africa; b Sequencing Core Facility, National Institute for Communicable Diseases, National Health Laboratory Service, Johannesburg, South Africa

## Abstract

Here, we report a draft genome sequence of *Mycobacterium peregrinum* obtained from a sputum sample of a South African HIV-infected patient with suspected pulmonary tuberculosis. The genome described here comprises 6,931,852 bp, revealing 66.2% G+C content, 6,808 coding sequences, and 81 RNA genes.

## GENOME ANNOUNCEMENT

*Mycobacterium peregrinum* is a nontuberculous mycobacterium that belongs to the *Mycobacterium fortuitum* complex. Although the complex members are not usually pathogenic for humans, they are opportunistic in that they can cause disease in people with disadvantageous conditions or who are immunocompromised (1, 2). A sputum specimen was submitted to the Supranational Tuberculosis (TB) Reference Laboratory, National Institute for Communicable Diseases, South Africa, as part of the National Drug Resistance Survey. The specimen was obtained from a 42-year-old female with a history of HIV, diagnosed clinically as a new TB case. Following sputum decontamination with NALC-NaOH, the time to positivity was 2 days on the Bactec MGIT 960 system (BD, Sparks, MD, USA). Molecular identification of the species using the GenoType *Mycobacterium* CM version 2.0 (Hain Lifescience GmbH, Nehren, Germany) identified the strain as *M. peregrinum*. We performed whole-genome sequencing to confirm the presence of *M. peregrinum*.

Genomic DNA was extracted from three independent liquid cultures using the NucliSENS easyMAG (bioMérieux, France); in brief, a 200-µl aliquot of the liquid culture was used as the input volume for DNA extraction using the generic protocol, with a final elution volume of 25 µl. Paired-end libraries were prepared using the Nextera XT DNA library kit, followed by 2 × 300-bp sequencing on an Illumina MiSeq (Illumina, San Diego, CA, USA). The paired-end reads were quality trimmed and *de novo* assembled using Qiagen CLC Genomics Workbench version 10 (Qiagen, The Netherlands). The assembly contains 216 contig sequences of longer than 500 bp and covers 6,931,852 bp, with a G+C content of 66.2% and an *N*_50_ of 124,779 bp. All contigs were then submitted to GenBank, where gene annotation was implemented using the NCBI Prokaryotic Genome Annotation Pipeline (PGAP) (3). The total number of 6,808 genes predicted by PGAP includes 6,582 protein-coding genes, 145 pseudogenes, and 81 RNA genes (75 tRNAs and 3 6RNAs). The annotation was further uploaded to Rapid Annotations using Subsystems Technology (RAST) for subsystems-based annotation (4–6). The RAST annotation assigned these genes into 436 subsystems, with a maximum number of genes associated with amino acids and derivative metabolism (15.99%).

### Accession number(s).

The draft genome sequence has been deposited at NCBI under the BioProject number PRJNA382269, BioSample number SAMN06702994, and accession number NCSV00000000.
